# Validity assessment for technical skills and stress management of the HelpMeSee ^®^ Manual Small Incision Cataract Surgery module

**DOI:** 10.1111/aos.70083

**Published:** 2026-01-31

**Authors:** Lea Dormegny, Marion Schaeffer, Nicole Neumann, Remi Yaïci, Lauriana Solecki, Gauthier Dechriste, Emilia Koestel, David Gaucher, Arnaud Sauer, Van Charles Lansingh, Anne Lejay, Nabil Chakfé, Tristan Bourcier

**Affiliations:** ^1^ Department of Ophthalmology, New Civil Hospital Strasbourg University Hospital, FMTS Strasbourg France; ^2^ Gepromed The Medical Hub for Patient Safety Strasbourg France; ^3^ HMS Jersey City New Jersey USA; ^4^ Instituto Mexicano de Oftalmología Santiago De Querétaro Mexico; ^5^ Department of Public Health Sciences University of Miami Miller School of Medicine Miami Florida USA; ^6^ Department of Ophthalmology and Visual Sciences John A. Moran Eye Center at the University of Utah Salt Lake City Utah USA; ^7^ Centro de Investigacion en Salud Poblacional instituto Nacional de Salud Publica Cuernavaca Mexico; ^8^ Department of Vascular Surgery and Kidney Transplantation Strasbourg University Hospital Strasbourg France

**Keywords:** cognitive load assessment, Messick framework, non‐technical skills, ophthalmology surgical training, stress management, surgical education, virtual reality simulation

## Abstract

**Purpose:**

To assess the validity of the HelpMeSee Manual Small Incision Cataract Surgery (MSICS) module as a virtual reality training tool for technical skills and stress management in ophthalmology.

**Methods:**

This prospective study enrolled 47 volunteer surgeons from five groups: four groups of eye surgeons with increasing experience (novice, junior, senior and expert) and a fifth group of experts from other specialties. Participants completed two standardized MSICS training runs on the HelpMeSee simulator. Performance scores, penalties and completion time were recorded. Ergonomics were assessed via the Rapid Upper Limb Assessment (RULA) score, and stress was evaluated subjectively and objectively using the State–Trait Anxiety Inventory‐Y (STAI‐Y) and the Analgesia Nociception Index (ANI) score. Data from the two runs were analysed and compared across groups.

**Results:**

Overall scores increased significantly from novice residents (32.4 ± 10.7 out of 72) to the expert ophthalmic surgeons (50.1 ± 9.41) (*p* < 0.001). Non‐ophthalmic experts had a lower mean score (16.8 ± 18.0). Total penalties, particularly in the second run, decreased with experience among eye surgeons, while experts from other specialties incurred the highest penalties. Time analysis did not differ between groups, as for RULA or STAI‐Y scores. The mean ANI score decreased with experience, suggesting higher stress levels in more experienced participants.

**Conclusions:**

The HelpMeSee MSICS module effectively differentiates surgical experience levels, confirming its validity as a tool for technical skills training. The ANI score demonstrated modified behaviour in expert surgeons, suggesting the simulator's potential for assessing non‐technical skills. These findings support the use of this virtual reality simulator for objective, skills‐based surgical education.

## 1 INTRODUCTION

Cataract surgery is the most common surgical procedure in ophthalmology, with 20 million operations performed worldwide each year (Gower et al., 2017). Different microsurgical procedures are performed worldwide, all requiring high visuospatial ability. Numerous training methods are currently proposed to learn this surgery, including written supports, surgical simulation (synthetic, animal or cadaver models and virtual reality simulators) (Ní Dhubhghaill et al., 2023), observation of patients' surgeries (video support or directly in the operating theatre) and the performance of supervised surgeries in the operating room (OR) (Dormegny, Lansingh, et al., 2024; Dormegny, Prior Filipe, et al., 2024; Osborne, 2007). On‐patient traditional apprenticeship models have been questioned due to the increased rate of surgical complications (from 3 to 50 times) in novice surgeons compared to experienced ones. This leads to increased morbidity for patients and higher costs for society (Carricondo et al., 2010; Johnston et al., 2010).

During the last decade, virtual reality simulation has gained more and more in popularity for surgical training, including ophthalmic surgeries. The availability of virtual reality simulation training facilities has increased in many university hospitals (Khan et al., 2023; Lansingh et al., 2022). Many studies report an optimized learning curve and a reduction of iatrogenic complications after virtual reality simulation training (Jacobsen et al., 2019; Lee et al., 2020; Lin et al., 2021; Nayer et al., 2020; Rothschild et al., 2020). Also, the safety of virtual reality simulation training supersedes any supervised on‐patient training program for cataract surgery (Dormegny, Lansingh, et al., 2024; Dormegny, Prior Filipe, et al., 2024). However, many different simulators currently exist and their accessibility and cost differ considerably, making them sometimes difficult to access for postgraduate students (Dormegny, Lansingh, et al., 2024; Dormegny, Prior Filipe, et al., 2024; Oseni et al., 2023).

The commercially‐available EyeSi^®^ simulator is the most widely used and studied, with approximately 1000 devices spread worldwide. It notably prepares surgeons for phacoemulsification technique, which is mostly performed in high‐income countries (HICs). Moreover, its high cost makes it difficult to access in low‐ and middle‐income countries (LMICs) (Dormegny, Lansingh, et al., 2024; Dormegny, Prior Filipe, et al., 2024), although they have the highest rates of cataract‐induced blindness (Khairallah et al., 2015; Singh & Strauss, 2015; Vision Loss Expert Group of the Global Burden of Disease Study & GBD 2019 Blindness and Vision Impairment Collaborators, 2024). In these countries, Manual Small Incision Cataract Surgery (MSICS) is mostly used, due to its better cost‐effectiveness profile with similar outcomes. Phacoemulsification is estimated to cost 1.4–4.7 times more than MSICS (Bernhisel & Pettey, 2020; Jaggernath et al., 2014; Khan et al., 2015). MSICS does not require phacoemulsification ultrasound probe but only instruments for manual lens removal. The HelpMeSee^®^ simulator (New York, NY) was developed for MSICS training and is integrated in LMICs training centres (Ahuja et al., 2025; Broyles et al., 2013; Lansingh & Nair, 2023). Despite its significant use, reports of HMS^®^ simulators' outcomes are sparse compared to those of the EyeSi^®^ simulator (Sankarananthan et al., 2022), with only one validated test for the assessment of MSICS cataract surgery skills using HelpMeSee^®^ simulator recently proposed by Hutter et al. (2023).

The Messick Validity Framework has previously been suggested as a valuable tool to assess the validity of a surgical simulation training (Thomsen et al., 2015a, 2015b). It proposes a five‐component framework exploring different sources of validity: Content, Response Process, Internal Structure, Relations with Other Variables and Consequences (Messick, 1989). The primary source of validity most often used in the literature is Relations with Other Variables, which corresponds to the association between assessment results and previous surgical experience (Nayer et al., 2020; Yaïci, Martinez‐Costa Pérez, et al., 2024; Yaïci, Poirot, et al., 2024). Validity assessment is typically related to the assessment of technical skills during simulation training (Nair et al., 2025; Thomsen et al., 2015a, 2015b), while surgical apprenticeship also relies on non‐technical skills. Indeed, those skills are fundamental for the management of intraoperative complications (Wood, Maqsood, Nanavaty, & Rajak, 2021; Wood, Maqsood, Zoutewelle, et al., 2021). Stress management is one major and well‐recognized non‐technical skills which is involved in the success of a surgical procedure (Miao & Xi, 2025). As for technical skills, stress management is closely related to the surgeons' level of experience when measured in the operating room (Richstone et al., 2010; Song et al., 2009). However, its assessment remains challenging with many different tools available (Sidhoum et al., 2023; Tam et al., 2025). Many questionnaires exist for the subjective assessment of stress and have been used for surgical procedures, including NASA‐TLX, SURG‐TLX and STAI notably (Hart, 2006; Hersen & Spielberger, 2003; Wilson et al., 2011). Objective measurement of stress level can include heart rate variability (HRV), electric conductivity of the skin (Schuetz et al., 2008), facial temperature (Pluyter et al., 2014), eye movements tracking (Tolvanen et al., 2022), blood or salivary samples analysis (Wetzel et al., 2011) and electroencephalography recording (Shafiei et al., 2025). Previous work demonstrated that HRV correlated with STAI measures in senior surgeons (Jones et al., 2015). Physical fatigue, which is an important part of the workload of one surgical task is difficult to assess during microsurgical procedure but has been previously evaluated using the Rapid Upper Limb Assessment (RULA) score (Dormegny et al., 2023; McAtamney & Nigel Corlett, 1993).

Finally, one can easily understand that expert surgeons better control their stress level during surgical performance in their respective specialty compared to young surgeons (Song et al., 2009). However, this is not systematically the case in the literature and might differ in the context of a simulated environment (Almukhtar et al., 2025). Stress management in experts needs further investigation. The evaluation of experts' stress management while performing a usual surgical procedure (i.e. out of their area of competence) might give new insight on their capacity to adapt to stressful conditions.

This study aimed to assess the construct validity of HelpMeSee^®^ simulator MSICS module for technical and non‐technical skills, specifically stress management, in surgeons of different levels of experience in cataract surgery, including non‐ophthalmic expert surgeons.

## 2 MATERIALS AND METHODS

This prospective, monocentric, descriptive, non‐randomized study was conducted within the Education Center of Gepromed in Strasbourg, France. Members of the Ethics Committee of the Faculty of Medicine in Strasbourg approved the study (CE‐2021‐6). Participants' inclusion was carried out from August 2022 to May 2023.

### 2.1 Participants

This study included volunteer eye surgeons of all levels of experience (i.e. from novice residents to expert surgeons) and other specialties, expert surgeons. All included participants signed a written and informed consent form regarding the use and publication of the collected data.

Forty‐seven surgeons were divided into five different groups. Eye surgeons were divided into four groups according to their level of experience in eye surgery. Group 1, or ‘novice residents’ group, for residents with <1 year of experience (i.e. starting their residency), Group 2, or ‘junior residents’ group, for residents with 1–4 years of experience (i.e. starting their practice in surgery), Group 3, or ‘senior surgeons’ group with 4–7 years (i.e. starting to practice surgery in autonomy), Group 4, or ‘expert eye surgeons’ group, with more than 7 years of practice (i.e. practicing surgery in autonomy for more than 2 years according to the French ophthalmology curriculum). Other specialties, expert surgeons (i.e. more than 7 years of experience in their surgical specialty) formed Group 5.

### 2.2 Virtual reality HelpMeSee ^®^ simulator

**FIGURE 1:**
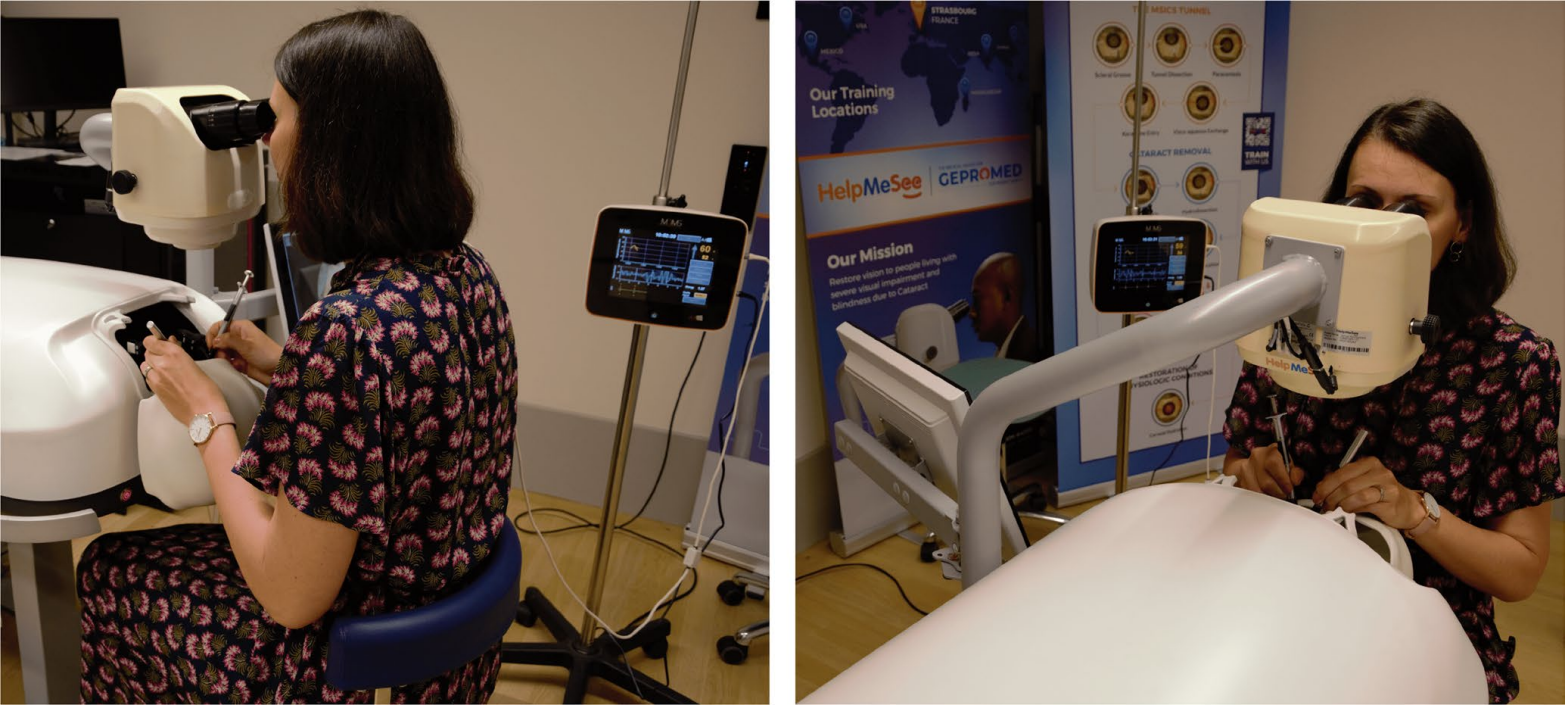
Set up of the simulation sessions. Participant performing the simulation session in a dedicated room on the HelpMeSee simulator with two electrodes (one on the right shoulder, the second at the apex of the heart) plugged to the Analgesia Nociception Index (ANI) monitor for instantaneous acquisition as a function of time over the whole simulation session.

The HelpMeSee^®^ (HMS) simulator (HelpMeSee foundation, Jersey City, New Jersey, United States) is a virtual reality simulator (Figure 1), offering a near‐realistic stereoscopic vision with high‐quality 3D images thanks to a microscope‐shaped design. Haptic feedback is provided through position and force sensors on both ophthalmic probes. The MSICS simulation‐based training course is divided into four modules: the sclero‐corneal tunnel course, the capsulorhexis and nucleus delivery course, the cortex removal and intra‐ocular lens implantation course, and the restoration of physical condition course (‘Manual Small Incision Cataract Surgery Simulation‐Based Training Course’, 2025). Each of these modules is also divided into several tasks. The scoring of each task, provided by the simulator's algorithm, is based on different metrics and different scales from one task to another. Moreover, negative penalty points can be awarded for significant errors such as entering in contact with the lens during incision or a zonular breakage higher than 50%.

### 2.3 Ergonomic and cognitive load assessment

Ergonomic investigations, more particularly participants' upper and lower body posture, were assessed using the Rapid Upper Limb Assessment (RULA) method (McAtamney & Nigel Corlett, 1993). Two ophthalmologists reviewed together the recorded session of each participant included in the study. They attributed a score for the position of the upper and lower arm, the wrist, the neck, the trunk and the legs in order to obtain a final score ranging from 1 to 7. One meant an acceptable posture while 7 indicated that investigation was required for change.

Cognitive load was objectively assessed with the ANI monitor V2 (MDoloris Medical Systems, France) (‘Our products—MDoloris Portefolio’, 2025) (Figure 1). Analgesia Nociception Index (ANI) reflects the activity of the parasympathetic nervous system. It is a mathematical derivative from the Heart Rate Variability (HRV) signal, which can be measured non‐invasively. This index is rated from 0 to 100, where 0 represents the maximum level of pain and thus stress, and 100 evaluates full wellbeing. The system used in the present study acquires the electrical signal through a self‐adhesive pair of electrodes placed on the participant and computes the ANI value over time (Le Gall et al., 2019; Rouby et al., 2024).

### 2.4 Study design

Simulation sessions were conducted in a dedicated room including the HMS simulator with the 1.1.2.12 software version and an ANI monitor V2. A short explicative document was given to all participants. Two electrodes plugged to the ANI monitor were placed on participants' right shoulder for the first and the apex of the heart for the second to acquire the instantaneous ANI as a function of time over the whole simulation session.

Prior to the simulation training session, two different subjective measures of cognitive load were realized: (1) the State–Trait Anxiety Inventory (‘Short STAI‐Y anxiety scales: validation and normative data for elderly subjects—PubMed’, n.d.; Gaudry et al., 1975) was used to measure participants' state anxiety (STAI‐YA) and trait anxiety (STAI‐YB) defined, for the first, as a transitory emotional response involving unpleasant feelings of tension and apprehensive thoughts and, for the second, as a personality trait referring to the likelihood of one individual experiencing state anxiety in a stressful situation; (2) they were asked to rate their pre‐session level of stress on a visual analogue scale from 0 to 10, where 0 was no stress at all and 10 indicated the maximum level of stress.

All participants completed then a recorded simulation session. After a warm‐up task (scleral cautery) to familiarize with the simulator interface, they performed nine tasks from the MSICS cataract surgery course presented with increasing difficulty, as follows: paracentesis and visco‐aqueous exchange (Task 1), IOL insertion and dialling (Task 2), hydrosuture (Task 3), hydrodissection (Task 4), nucleus prolapse (Task 5), cortex removal (Task 6), tunnel dissection (Task 7), capsulorhexis (Task 8) and nucleus delivery (Task 9). Levels of difficulty were weighted by experts surgeons (T.B. and J.‐M.A.) and as per the literature (Ní Dhubhghaill et al., 2023). Each task was preceded by a short instructional film. Then the task had to be performed twice in a row (Run 1 and Run 2).

**FIGURE 2:**
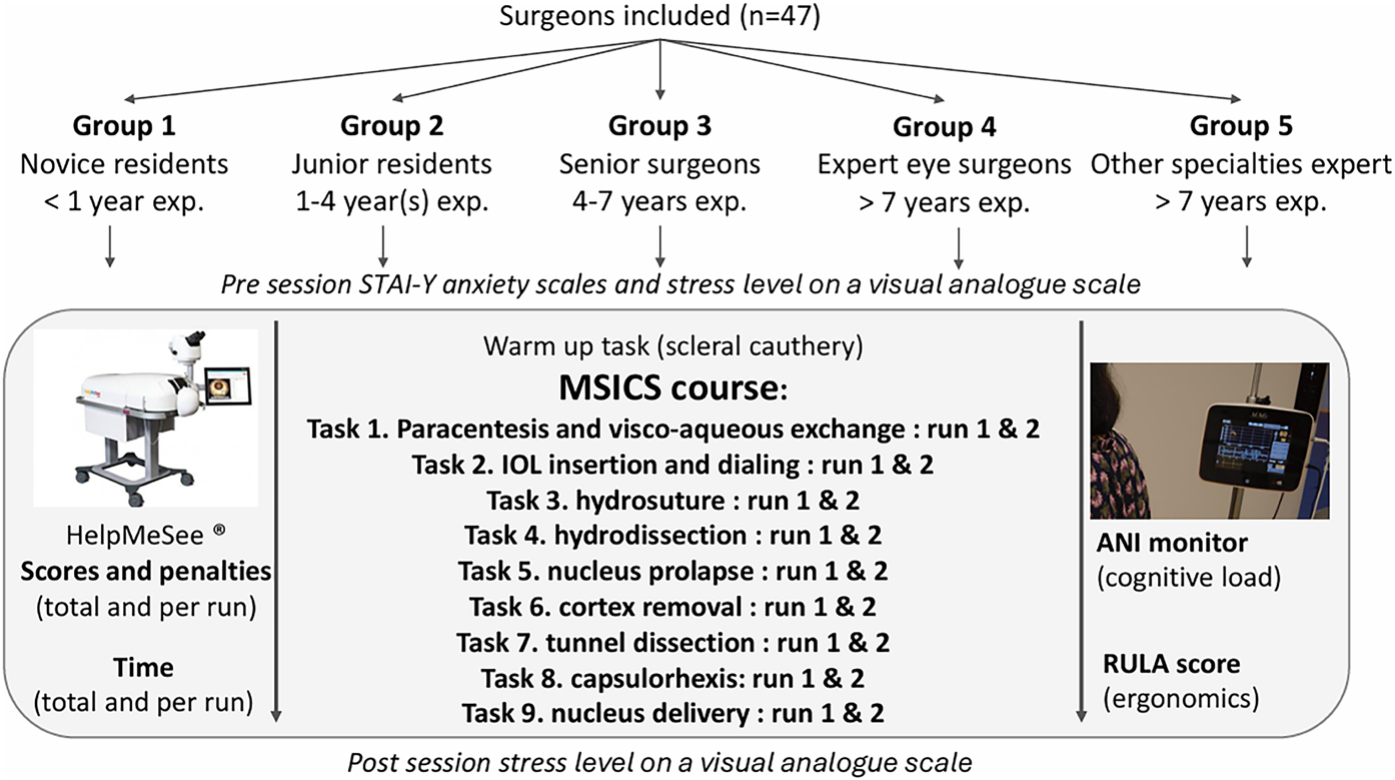
Design of the study. Participants were categorized into 5 groups based on their level of surgical experience (exp.) and surgical specialty. The procedure involved a pre‐session subjective anxiety assessment (using the State–Trait Anxiety Inventory‐Y [STAI‐Y] and a visual analogue scale), followed by the Manual Small Incision Cataract Surgery (MSICS) course. Each task within the course was performed twice (Run 1 and Run 2). Measurements (detailed inside the grey box, including the Rapid Upper Limb Assessment [RULA] and Analgesia Nociception Index [ANI]) were conducted throughout the session. A final, post‐session subjective anxiety assessment (visual analogue scale) was then performed.

At the end of the simulation session, participants were asked to provide a subjective self‐evaluation of their post‐session level of stress on the same visual analogue scale from 0 to 10. The study design is illustrated in Figure 2.

### 2.5 Outcome metrics

Construct validity of the HMS simulator MSICS module was assessed by comparing the overall total score and accumulated penalties between the study groups. Scores and penalties were extracted directly from the simulator (scoring details in Appendix S1). The overall total score and cumulative penalties were also calculated for each run and compared between the study groups.

Performance time was calculated as the cumulative time to end each of the nine tasks for one given run. Cognitive load was objectively assessed by the ANI. Initial ANI value, the mean and standard deviation of the ANI during the tasks were measured. These parameters correspond to the mean ANI over the last 3 minutes preceding the warm‐up task, the mean ANI over the whole run and session and the standard deviation over the whole run and session, respectively. Subjective measure of the cognitive load was assessed using the STAI and visual analogue scale (pre‐ and post session). Ergonomics was assessed using the RULA score, measuring the position of the different joints of the upper and lower body during a surgical task by two different assessors (M.S. and G.D) (McAtamney & Nigel Corlett, 1993).

### 2.6 Statistical analysis

Statistical data were expressed as percentages or mean and standard deviations to describe the normally distributed variables, or median and interquartile range for not‐normally distributed variables. Normality was assessed with the Shapiro–Wilk test. Comparisons of the computed variables between groups were developed using the Kruskal–Wallis test for non‐normally distributed variables and the ANOVA test for normally distributed variables. Hypothesis were considered statistically significant when the *p*‐value was lower than 0.05. Statistical analysis was performed with functions from the Python *scipy.stats* module.

## 3 RESULTS

### 3.1 Participant characteristics

Over the 47 enrolled participants, 9 residents were assigned to the novice residents group (Group 1), 12 subjects in the junior residents group (Group 2), 9 in the senior surgeons group (Group 3), 9 surgeons in the expert ophthalmic surgeons group (Group 4) including 7 phacoemulsification experts and 2 phacoemulsification and MSICS experts, and 8 in the other specialties expert surgeons group including 4 vascular surgeons, 3 neurosurgeons and 1 orthopaedic surgeon (group 5). Demographic and characteristic details of the studied groups are shown in Table 1.

**TABLE 1:** Demographic and baseline characteristics of the studied groups. IQR, interquartile range.

	Novice—Group 1	Junior—Group 2	Senior—Group 3	Experts—Group 4	Other surgical specialties—Group 5
Number of participants	9	12	9	9	8
Strong hand, % (*n*)					
Ambidextrous	0% (0)	8.33% (1)	0% (0)	0% (0)	0% (0)
Right	100% (9)	83.3% (10)	88.9% (8)	77.8% (7)	100% (8)
Left	0% (0)	8.33% (1)	11.1% (1)	22.2% (2)	0% (0)
Sex, % (*n*)					
Women	44.4% (4)	75.0% (9)	22.2% (2)	0.00% (0)	62.5% (5)
Men	55.6% (5)	25.0% (3)	77.8% (7)	100% (9)	37.5% (3)
Age, median (IQR)	24.5 (24–25)	27 (27–27.5)	30 (30–31.5)	46 (40–65.5)	38 (34–43)

### 3.2 Score and time analysis

**FIGURE 3:**
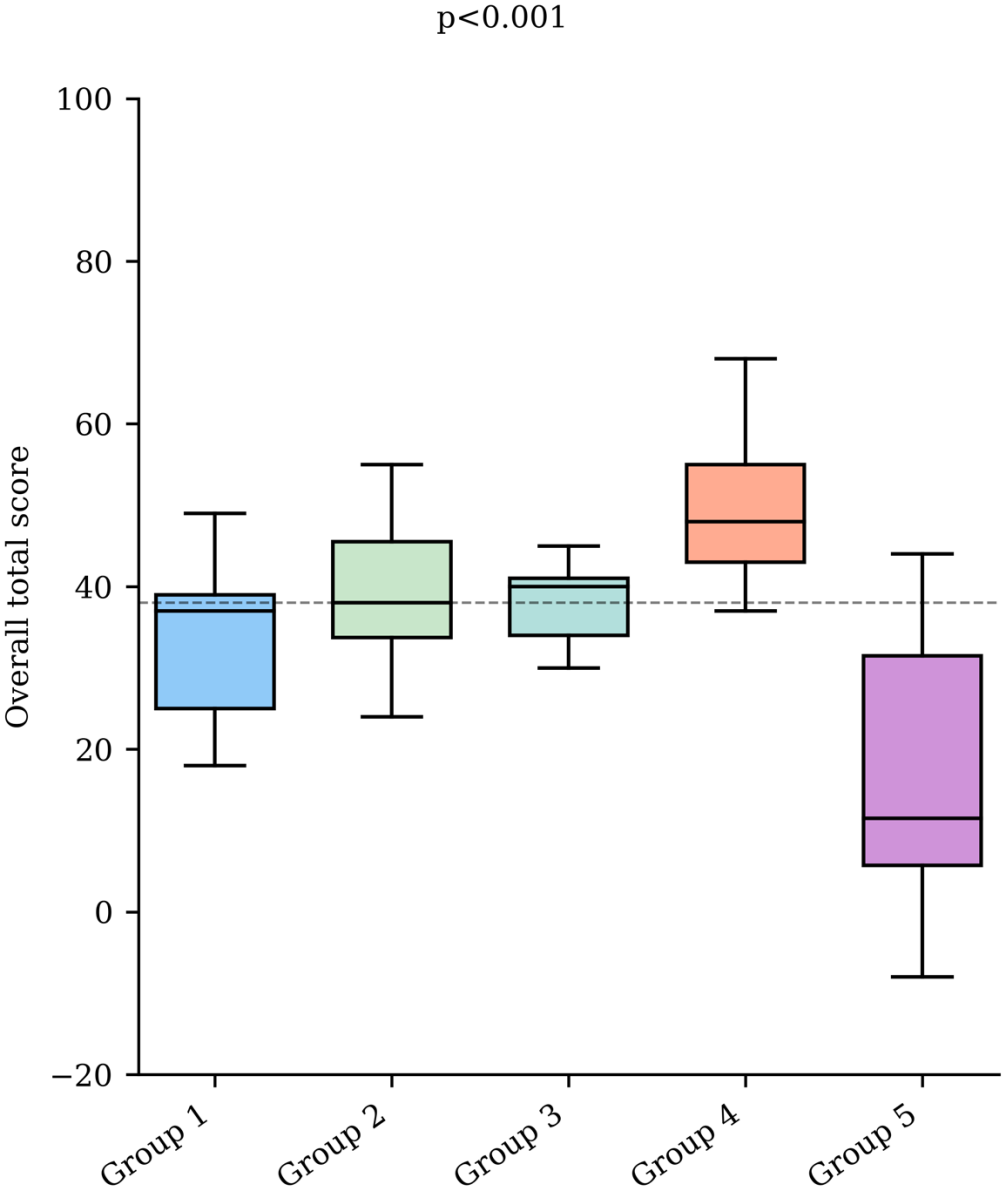
Overall performance score for each of the 5 study groups. The overall total score progressively and significantly increased (*p* < 0.001) from the novice residents group (32.4 ± 10.7 out of 72) to the expert ophthalmic surgeons group (50.1 ± 9.41), while other specialties' expert surgeons presented a lower mean score of (16.8 ± 18.0).

The overall total score progressively and significantly increased (*p* < 0.001) from the novice residents' group (32.4 ± 10.7 out of 72) to the expert ophthalmic surgeons group (50.1 ± 9.41), while non‐ophthalmic expert surgeons presented a lower mean score of (16.8 ± 18.0). Figure 3 depicts the distribution of the overall score for each group. Penalties were also lower for ophthalmology experts compared to less experienced ophthalmologists (*p* < 0.001).

Scores and penalties analysis and comparisons between groups are detailed in Table 2. The total score of Run 1 and Run 2 both significantly differed between groups (*p* = 0.02 and *p* < 0.001, respectively), non‐ophthalmic experts also showing a lower level than the other groups. When comparing Run 1 to Run 2, all participants improved between the two runs, regardless of the group to which they belonged. Total penalties scores only differed between groups for Run 2 (*p* = 0.007), with non‐ophthalmic surgeons receiving the highest number of penalties of this run. Penalties for Run 1 did not differ between groups.

**TABLE 2:** Technical skills (score and time) analyses compared for each group.

	Novice—Group 1	Junior—Group 2	Senior—Group 3	Experts—Group 4	Other surgical specialties—Group 5	*p*‐value
Score analysis, mean (SD)						
Overall total score	32.4 (9.96)	38.9 (9.14)	38.0 (5.16)	50.1 (8.87)	16.8 (16.8)	<0.001*
Total score Run 1	15.2 (6.29)	18.1 (7.11)	18.0 (3.53)	23.2 (4.94)	6.63 (11.2)	0.02*
Total score Run 2	17.2 (5.33)	20.8 (5.86)	20.0 (3.97)	26.9 (5.28)	10.13 (8.39)	<0.001*
Overall total penalities	16.4 (5.66)	11.3 (5.20)	11.3 (3.65)	6.33 (5.56)	22.5 (10.6)	<0.001*
Total penalities Run 1	8.11 (3.28)	6.83 (4.83)	5.56 (1.95)	3.67 (3.09)	11.6 (7.60)	0.053
Total penalities Run 2	8.33 (3.65)	4.50 (3.99)	5.78 (3.71)	2.67 (3.59)	10.9 (5.01)	0.007*
Time analysis (min), mean (SD)						
Total Time	39.2 (4.90)	38.8 (7.16)	37.7 (4.75)	33.9 (8.99)	39.9 (7.42)	0.434
Time Run 1	22.8 (2.09)	21.5 (3.68)	21.4 (2.98)	19.2 (5.66)	20.9 (2.83)	0.419
Time Run 2	16.4 (3.07)	17.3 (3.95)	16.3 (2.49)	14.7 (3.65)	18.9 (5.67)	0.357

*Note*: Overall total score, total score for Run 1 and total score for Run 2 significantly increased with the level of experience in eye surgeons groups, while other surgical specialties experts presented with the lowest score. Overall total penalties and total penalties for Run 2 significantly decreased with the level of experience in eye surgeons groups, while other surgical specialties experts presented with the highest penalties scores. Time analysis did not differ between groups.

Abbreviation: SD, standard deviation.

* Statistically significant.

Several tasks showed different results between groups as this was the case for Tasks 1 (paracentesis and visco‐aqueous exchange) and 6 (cortex removal) (*p* = 0.007 for the Run 1 of Task 1 and *p* = 0.007 for the second run of task 6). On the other hand, task 7 (tunnel dissection) was similar between groups (Appendix S2).

Concerning time to perform the tasks, experts' surgeons tended to be faster than novice residents; however, results did not reach significance (Table 2). Performance time improved between both runs in all groups, although the improvement did not correlate with participants' group of experience (*p* = 0.277). On average, each participant reduced his/her time by 4.48 ± 3.45 minutes from Run 1 to Run 2.

Despite an unclear distinction in terms of time between all the groups, there was a linear correlation between time to perform the simulation session and acquired total score. Participants with the highest total scores were also the fastest (*p* = 0.017). Although when isolating Run 1 and Run 2, this correlation remained significant only for Run 1 (*p* = 0.047).

### 3.3 Ergonomic and cognitive load

Ergonomics and cognitive load assessments are detailed in Table 3. RULA score was similar between groups. The average score for all participants was 2.43 ± 0.56 out of 7, meaning that the posture was acceptable or possibly that a slight change is required.

**TABLE 3:** Ergonomics and objective and subjective stress management assessments are presented for each group.

	Novice—Group 1	Junior—Group 2	Senior—Group 3	Experts—Group 4	Other surgical specialties—Group 5	*p*‐value
RULA score, mean (SD)	2.40 (0.49)	2.71 (0.45)	2.57 (0.49)	2.00 (0.53)	2.50 (0.50)	0.214
**STAI‐Y analysis, % (*n*)**						
Level of anxiety Y1						0.778
No or little anxiety	62.5% (5)	75.0% (9)	55.6%(5)	77.8% (7)	75.0% (6)	
More anxious than average	0.00% (0)	8.33% (1)	0.00% (0)	0.00% (0)	12.5% (1)	
Above average	37.5% (3)	16.7% (2)	33.3% (3)	11.1% (1)	12.5% (1)	
Very severe anxiety	0.00% (0)	0.00% (0)	11.1% (1)	11.1% (1)	0.00% (0)	
Personality Y2						0.661
Not anxious	50.0% (4)	75.0% (9)	55.6% (5)	66.7% (6)	75.0% (6)	
Anxious	50.0% (4)	25.0% (3)	33.3% (3)	33.3% (3)	12.5% (1)	
Very anxious	0.00% (0)	0.00% (0)	11.1% (1)	0.00% (0)	12.5% (1)	
Stress assessment						
Level of stress before, median (IQR)	1.50 (0.75–2.25)	1.50 (0.75–2.25)	1.00 (0.00–3.00)	1.00 (0.00–3.00)	3.00 (1.75–5.00)	0.502
Level of stress after, median (IQR)	3.00 (1.00–4.25)	3.00 (0.50–3.50)	1.00 (0.00–2.00)	1.00 (0.00–3.00)	4.00 (2.75–4.00)	0.437
Initial ANI, mean (SD)	70.8 (10.1)	75.0 (12.5)	62.4 (9.37)	67.2 (10.5)	65.4 (12.5)	0.203
Mean ANI, mean (SD)	73.8 (6.66)	76.0 (11.7)	63.8 (7.38)	63.6 (9.84)	65.9 (8.04)	0.023*
Standard deviation ANI, mean (SD)	11.4 (2.52)	10.1 (1.89)	9.51 (1.22)	10.5 (4.27)	10.3 (1.81)	0.486

*Note*: Rapid Upper Limb Assessment (RULA) score for ergonomics assessment did not differ between groups. STAI‐Y subjective analyses for anxiety state and trait did not differ between groups. Mean ANI significantly decreases with the level of experience, regardless of the surgical specialty, meaning that the more experienced participants presented the highest levels of stress using this assessment method.

Abbreviations: IQR, interquartile range; RULA, Rapid Upper Limb Assessment; SD, standard deviation; STAI‐Y, State‐Trait Anxiety Inventory‐Y.

* Statistically significant.

Cognitive load assessment included subjective assessments (STAI‐YA, STAI‐YB and self‐assessment on a pre‐ and post‐ session visual analogue scale) and objective measurements (ANI V2 monitor).

Types of personality and anxiety states using STAI were quite uniformly distributed within each group. Self‐assessment of stress showed that non‐ophthalmic expert surgeons tended to feel more stressed than ophthalmologic surgeons, regardless of their surgical level (*p* = 0.071 before the simulation session and *p* = 0.147 afterwards). Only novice and junior residents (Groups 1 and 2) had a higher level of stress after the simulation session.

**FIGURE 4:**
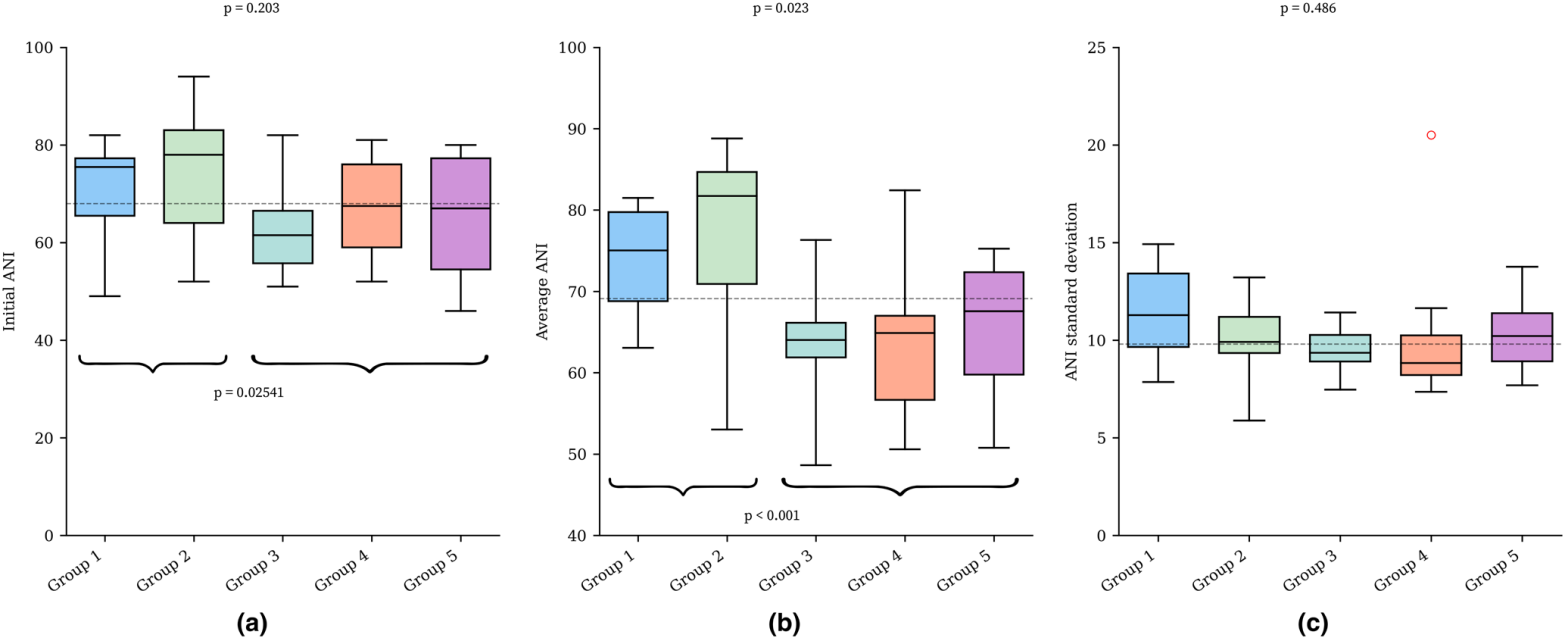
Objective stress management assessment using Analgesia Nociception Index (ANI) monitor. Data acquired for both runs (1 and 2) are displayed for each of the studied groups. (a) Initial ANI tends to decrease with the degree of expertise of the participants, but statistical comparisons are not significant (*p* = 0.203). However, when separating participants into two different groups of level of experience (i.e. Groups 1 and 2 pulled together above the dotted line representing lower levels of experience; and Groups 3, 4 and 5 pulled together underneath the dotted line representing higher levels of experience). Comparison between these two groups shows that initial ANI is lower in groups of high levels of experience compared to lower levels of experience groups (64.8 vs. 73.3; *p* = 0.025, Student t‐test), meaning that higher experienced surgeons have more objective manifestations of stress initially. (b) Mean ANI significantly decreases, meaning that stress level increases, with the degree of experience of the participants (*p* = 0.023). When separating participants into the same two different groups of level of experience (see braces), mean ANI was lower in groups of high levels of experience compared to lower levels of experience groups, confirming that higher experienced surgeons have more objective manifestations of stress initially (*p* < 0.001). (c) Standard deviation of ANI signal did not differ between groups.

Based on the data acquisition from the ANI V2 monitor, the initial stress level, the mean and standard deviation ANI values over both runs have been computed for each participant. Distribution of these values according to the different groups is represented in Figure 4. The mean ANI over the simulation session tended to be increasingly important with participants' level of expertise, independently of the surgical specialty (*p* = 0.023). Participants were pulled together in two groups, differentiated by their level of expertise (Groups 1 and 2 for low level and Groups 3, 4 and 5 for higher level of expertise). With this new classification, higher level participants were much more stressed than low level participants: *p* = 0.025 for the initial ANI and *p* < 0.001 for the mean ANI. The standard deviation of the ANI signal did not differ between groups. Personality traits (STAI‐Y1 and STAI‐Y2) did not correlate with the initial ANI.

## 4 DISCUSSION

This study aimed to assess the construct validity of the MSICS module of HelpMeSee^®^'s virtual reality surgical simulator for technical skills and stress management, in surgeons of different levels of experience in cataract surgery and non‐ophthalmic expert surgeons.

### 4.1 Technical skills assessment

Overall total score (both runs) and total penalties (Run 2) significantly differed between groups. Performance score increased and number of penalties decreased with the level of experience of the study groups, demonstrating validity evidence of the MSICS module according to Messick Validity Framework (‘relations to other variables’ plan) (Messick, 1989). Two other studies reported validity evidence of the MSICS module (Hutter et al., 2023; Nair et al., 2022). Nair et al. established content validity of the HelpMeSee^®^ simulator, based on experts' opinion and corresponding to the Content component of the Messick Validity Framework. Thirty‐five MSICS experts were interrogated on the validity of the sclero‐corneal tunnel construction course compared to their personal on‐patient MSICS surgery experience (Nair et al., 2022). Hutter et al. propose a thirty‐metric test, selected from the 11 steps of the HelpMeSee^®^ MSICS course based on their discriminative ability. Going one step further, they established one pass‐fail score for this particular test (20/30), corresponding to the Consequences component of the Messick Validity Framework. Additionally, they assessed the reliability of the test (relevant to the Internal Structure component) by calculating an Interclass Correlation Coefficient (ICC) (Messick, 1989).

As chosen by Hutter et al., validity assessment of technical skills (scores and penalties) was based on the simulator scoring system in the present study. This allows objective and reproducible rating of performances (Thomsen et al., 2015a, 2015b). Nine steps from the 11 proposed by the simulator course were selected. All metrics from each step were included to ensure easier replication of the training session. Considering the previous content validity assessment of the sclero‐corneal tunnel construction course (Nair et al., 2022) and MSICS expert surgeons (T.B. and J.‐M. A.) review, ‘creating scleral groove’ and ‘entering the anterior chamber with keratome’ steps were excluded as considered insufficiently discriminant (the first one being insufficiently realistic and the second one too hard to achieve). This review and selection was realized considering the participation of non‐ophthalmic surgeons (‘other specialties experts’ group) in order to avoid making the task too difficult and their runs too long and tiring.

Scores for each step were also compared between groups. Differences were found in both runs for Tasks 1 (paracentesis and visco‐aqueous exchange) and 6 (cortex removal) (Appendix S2). Paracentesis and visco‐aqueous exchange were considered the most realistic step in Nair et al.'s study (Nair et al., 2022), comforting these results. Hutter et al. considered the size of the paracentesis and the management to avoid contact with the iris particularly discriminant to be included in their test (Hutter et al., 2023). On the other hand, these steps are considered relatively simple by ophthalmology surgeons, notably residents (Ní Dhubhghaill et al., 2023), and one might have considered them too easy to be discriminant. In the present study, paracentesis and visco‐aqueous exchange (Task 1) were the first to be performed. One might think that acclimatization to the simulator might have interfered with the results, notably for novices and non‐ophthalmic surgeons. However, differences between groups remained after run number 2, supporting this first result. Task 6 (cortex removal), also considered one relatively simple step by cataract surgeons (Ní Dhubhghaill et al., 2023), requires the use of an irrigation/aspiration probe which might be challenging for unfamiliar surgeons, making a significant difference between groups.

Tasks 3 (hydrosuture), 5 (nucleus prolapse) and 7 (tunnel dissection) performance scores were similar between groups, suggesting these steps were less discriminant. Notably, Group 4 included experts in phacoemulsification (78%) and experts in phacoemulsification and MSICS (22%). As Tasks 1 and 6 are common between phacoemulsification and MSICS procedures, these might have been easier to perform for phacoemulsification experts, while Tasks 5 and 7 are specific to MSICS, making them more challenging even for the phacoemulsification expert surgeons. Task 3 (hydrosuture) might have been quite easy to perform for all participants as it requires only one instrument, making it less discriminant.

In a previous validity study for the HelpMeSee phacoemulsification course, significant differences between junior and senior participants were reported for capsulorhexis and quarter removal tasks (Yaïci, Martinez‐Costa Pérez, et al., 2024; Yaïci, Poirot, et al., 2024). While quarter removal is not part of the MSICS procedure, the capsulorhexis task scores also significantly differed between groups for Run 2 in the present study.

In the present study, time to perform surgical tasks did not differ between groups. However, the duration of the surgery negatively correlated with the performance score of the participants, meaning that surgery quality decreased with time (Table 2). Indeed, it has been shown that surgery duration is associated with an increased risk of complications (Cheng et al., 2018; Guidolin et al., 2022). In other simulated environments, expert eye surgeons operate faster than less experienced surgeons (Dormegny et al., 2023). The ‘step by step’ approach of the present training program might explain why we did not find significant differences between the study groups. Assessment of time to perform the entire procedure continuously might be more discriminant between surgeons for different levels of expertise.

### 4.2 Stress management assessment

Stress management in surgeons is directly related to the occurrence of per operative errors (Arora et al., 2010; Hassan et al., 2006). Indeed, stress management significantly impacts both technical and non‐technical skills (Tam et al., 2025). It is even considered one particular non‐technical skill, working in close relation with other skills in this category (i.e. teamwork, communication, situation awareness and leadership) (Chan et al., 2024; Wood, Maqsood, Nanavaty, & Rajak, 2021; Wood, Maqsood, Zoutewelle, et al., 2021). It has been demonstrated that the level of stress and its perception directly modify its impact on performance. Thus, moderate, controlled and well‐tolerated levels of stress increase the level of concentration and motivation of the surgeon and have positive effects on surgical performance. While excessive levels of stress impair concentration, reduce decision‐making capacity and increase the risk of errors and surgical complications (Yerkes & Dodson, 1908).

This could explain why in the present study, objective stress measurements by ANI showed higher levels of stress in the most experienced surgeons compared to those with lower experience. Moreover, in experienced surgeons, we observed less variation of the mean ANI during the procedure, probably indicating a constant, moderate level of stress with adapted reactions to the progress of the surgery. Other specialties, expert surgeons better managed their stress compared to novice ophthalmologists, which comforts the hypothesis that stress management is related to the seniority of a surgeon more than surgery routine. Indeed, surgeons from this group all discovered the eye surgery simulator interface for the first time, yet were able to manage their stress in a different way than novice surgeons. The same observation was recently reported for endovascular surgery performance on a simulator. ANI monitor showed higher levels of stress in experienced surgeons compared to naïve residents (Rouby et al., 2024).

Subjective assessments demonstrate lower levels of stress in higher experienced during surgical performance surgery, either in the operating room or in a simulated environment happen (Anschuetz et al., 2019; Kennedy‐Metz et al., 2020; Mohamed et al., 2014). Interestingly, in the present study, other specialties' expert surgeons tend to feel more stressed, according to subjective assessment scale, than ophthalmologic surgeons of all levels of experience. This might reflect performance anxiety in the context of a completely unknown environment for non‐ophthalmic surgeons. Even novice and junior ophthalmologists were less anxious about their performance: these groups were probably less aware of the possible consequences of their performances on real patients, being also less able to project themselves in an operating theatre. Interestingly, subjective stress levels in these two groups (groups 1 and 2) increased after the simulation session. Although simulation training helps increase confidence in surgical performance when used to this end (Staropoli et al., 2018), it is possible that novice and junior ophthalmologists experienced the presented simulated environment differently. As the present study aimed to assess the validity of MSICS training course, exposure to the simulator tasks was short (two runs of performance). This quick confrontation to the role of a first operator surgeon, without any reattempt possibility in case of surgical error, might have increased the degree of stress in young surgeons, giving them a small overview of what can happen in real OR environment. If they had been allowed to continue training (Wood, Maqsood, Nanavaty, & Rajak, 2021; Wood, Maqsood, Zoutewelle, et al., 2021), the stress level would probably have decreased, finally.

### 4.3 Ergonomic assessment

Ergonomic assessment of a surgical simulation device needs to be considered. Indeed, musculoskeletal disorders have been reported in eye surgeons (Kaup et al., 2018), indicating that monitoring of surgeons' position should be performed during surgical training. The RULA score, which has been designed to evaluate the surgeon's posture while seated (McAtamney & Nigel Corlett, 1993), did not differ between groups. Indeed, in the simulated and reproducible environment proposed here, head, trunk, upper limbs and wrist positions of the participants were less prone to change during the procedure. Also, participants could reposition after each performed task which might differ during an entire MSICS procedure. Similar results were observed on the different simulation module using synthetic eyes for corneal stitches performance (Dormegny et al., 2023). Other three‐dimensional recording of finer movements, as finger and hand motions during ophthalmic surgery might be more adapted (Saleh et al., 2006).

### 4.4 Other specialties expert surgeons assessment

Interestingly, other specialties surgeons presented with the lowest performance scores (total score for both runs and total penalties for Run 2) compared to other groups, including the novice eye surgeons group. To our knowledge, this is the first report of a crossed‐specialties simulation training program in surgery. These results might be explained by the relative lack of theoretical knowledge and routine ophthalmology practice in this particular group. This includes notably slit lamp and fundus examinations, observation in the operating room and participation to other simulation training sessions (synthetic or animal eyes). Indeed, eye surgeons start experiencing these practices early in their curriculum (Deuchler et al., 2022, 2023; Ní Dhubhghaill et al., 2023) and are highly encouraged by national authorities to do so (Dormegny, Lansingh, et al., 2024; Dormegny, Prior Filipe, et al., 2024; Filipe et al., 2025). Indeed, eye–hand coordination is continuously trained during eye examination and greatly differs from the general medical examination performed by other specialties surgeons. Completely different surgical environments are also experienced in these specialties. Excepting selected procedures, surgeries are performed in a standing position, without an operating microscope. Also, surgical instruments used are bigger than those in ophthalmology (Calvanese et al., 2025; Gardezi et al., 2025; Tanamal et al., 2025). Changing these parameters forced other specialties surgeons to learn new technical skills. They presented with a similar learning curve (from Run 1 to Run 2) compared to the other study groups, meaning that this new activity truly challenged them. However, their stress management was similar to expert eye surgeons, suggesting that non‐technical skills are transferable to multiple environments. Indeed, a high level of non‐technical skills equals a high capacity of adaptation in an unusual, possibly stressful, situation as this was the case here.

### 4.5 Limitations

This study included a small number of participants (*n* = 47), although it is relatively high compared to other surgical simulation studies (Bergqvist et al., 2014; Jaud et al., 2020). Expertise level definition was based on the French model of surgical training where surgery is taught during residency, then surgical activity drastically increases during fellowship and even more after fellowship (>7 years) (Yaïci, Filipe, et al., 2025; Yaïci, Schiefelbein, et al., 2025). This teaching method differs from other countries, as surgical training remains very heterogeneous, even between European countries (Ní Dhubhghaill et al., 2023; Yaïci, Filipe, et al., 2025; Yaïci, Martinez‐Costa Pérez, et al., 2024; Yaïci, Poirot, et al., 2024; Yaïci, Schiefelbein, et al., 2025).

Metrics from the HelpMeSee^®^ simulator ensured scoring objectivity, although the extent of the scoring system is relatively short as binary scores are used for some tasks (Appendix S1). More detailed scoring systems could be used to better discriminate participants of different levels of experience, as proposed in other simulators' scoring systems (Jaud et al., 2020; Nayer et al., 2020).

Expert eye surgeons (Group 4) included experts in phacoemulsification (78%) and experts in both MSICS and phacoemulsification (22%). In France, phacoemulsification is the most largely implemented technique and exclusive MSICS experts are very rare, making the distinction between both surgical practices difficult in our study (Malot et al., 2011). Although, we considered that including experts in phacoemulsification and/or MSICS in Group 4 was reasonable as (1) both surgeries have many steps in common (6/9 [67%] in the present study) leading to small differences between phacoemulsification experts and exclusive MSICS experts and (2) phacoemulsification experts go on humanitarian missions and MISCS should be part of their surgical repertory, making their performance on the MSICS course meaningful (Berrones et al., 2025).

Surgical tasks were presented in increasing order of difficulty and not from the start of the procedure. Although this steps away from the real MSICS sequence, this choice was made to ensure the following: (1) progressive adaptation to the simulator interface, notably for other specialties experts, increasing performance comparability with other groups; (2) alignment with ‘on‐patient’ apprenticeship model, where the learner is asked to start ‘from the end’ of the surgery, assuming that these steps are at lower risk of complications for the patient. Also, the performance of a second run helped confirm findings from the first run after participants were more comfortable with the simulator interface.

## 5 CONCLUSION

The present study demonstrates validity evidence of a multiple MSICS tasks program from the HelpMeSee^®^ virtual reality simulator MSICS course, which is easily replicable. Particular emphasis is made on the assessment of surgeons' stress management capacities, which were different between non‐experienced surgeons and experienced ones, regardless of their surgical specialty. Expert surgeons from other specialties presented with similar behaviour of stress management compared to eye expert surgeons, although they had poorer performance than all other eye surgeons, regardless of their experience. Non‐technical skills are remaining competencies of experienced surgeons which they can gather in unusual and stressful situations to stay in control. Further validity studies should take non‐technical skills assessment into account in a simulated environment, as those seem to mirror one surgeon's previous experience in the operating room.

## FUNDING INFORMATION

No funding source was used for this study. One of the authors of the manuscript (V.C. Lansingh) is currently employed by HelpMeSee, Inc. (Jersey City, New York).

## Supporting information


Appendix S1.

Appendix S2.
