# Supplementary material for: Validity assessment for technical skills and stress management of the HelpMeSee ® Manual Small Incision Cataract Surgery module
Source: Acta Ophthalmol. 2026 Jan 31;104(5):e503–15. doi: 10.1111/aos.70083 (PMC13353712; doi:10.1111/aos.70083)
Supplement: Supplementary file 1 — Appendix S1. [file AOS-104-e503-s002.docx]

| Tasks | Assignment items | Score | Penalty | Total score |
| --- | --- | --- | --- | --- |
| Making a Paracentesis and Injecting Viscoelastic | Contact with the iris | 1 |  | 7 |
|  | Endothelial touch | 1 |  |  |
|  | Contact with lens | 1 |  |  |
|  | Contact with the iris | 1 |  |  |
|  | Endothelial touch | 1 |  |  |
|  | Size of the paracentesis (mm) | 1 |  |  |
|  | Contact with lens | 1 |  |  |
| Inserting and Dialing the IOL | IOL completely inside the capsular bag | 1 |  | 4 |
|  | Lost IOL | 1 |  |  |
|  | Zonular breakage (%) | 1 |  |  |
|  | Endothelial touch | 1 |  |  |
|  | Zonular breakage more than 50% |  | -2 |  |
|  | Zonular breakage more than 50% |  | -4 |  |
| Hydrating the Paracentesis Site | Endothelial touch | 1 |  | 2 |
|  | Contact with the iris | 1 |  |  |
| Performing Hydrodissection and Hydroprolapse | Partial hydrodissection | 1 |  | 5 |
|  | Zonular breakage (%) | 1 |  |  |
|  | Cortical hydration | 1 |  |  |
|  | Contact with the iris | 0 |  |  |
|  | Complete hydrodissection | 1 |  |  |
|  | Endothelial touch | 1 |  |  |
|  | Zonular breakage more than 50% |  | -6 |  |
| Dislocating the Nucleus | Endothelial touch | 1 |  | 3 |
|  | Contact with the iris | 1 |  |  |
|  | Zonular breakage (%) | 1 |  |  |
|  | Zonular breakage more than 50% |  | -4 |  |
| Removing the Cortex | Amount of residual cortex (%) | 1 |  | 6 |
|  | Zonular breakage more than 50% |  | -7 |  |
|  | Location of residual cortex | 1 |  |  |
|  | Posterior capsular rupture |  | -7 |  |
|  | Iris aspiration | 1 |  |  |
|  | Capsular aspiration | 1 |  |  |
|  | Endothelial touch | 1 |  |  |
|  | Zonular breakage (%) | 1 |  |  |
| Dissecting a Tunnel | Premature entry |  | -3 | 2 |
|  | Length of the inner tunnel limit (mm) | 1 |  |  |
|  | Laceration of the outer wall |  | -3 |  |
|  | Length of the tunnel (mm) | 1 |  |  |
|  | Perforation of the outer wall (button holes) |  | -3 |  |
|  | Uveal prolapse |  | -3 |  |
| Making a Capsulorrhexis with Utrata Forceps | Maximum size of capsulorrhexis (mm) | 1 |  | 5 |
|  | Runout |  | -6 |  |
|  | Minimum size of capsulorrhexis (mm) | 1 |  |  |
|  | Contact with the iris | 1 |  |  |
|  | Zonular breakage more than 50% |  | -6 |  |
|  | Zonular breakage (%) | 1 |  |  |
|  | Endothelial touch | 1 |  |  |
| Delivering the Nucleus | Iris pull | 1 |  | 2 |
|  | Endothelial touch | 1 |  |  |
