# Supplementary material for: Validity assessment for technical skills and stress management of the HelpMeSee ® Manual Small Incision Cataract Surgery module
Source: Acta Ophthalmol. 2026 Jan 31;104(5):e503–15. doi: 10.1111/aos.70083 (PMC13353712; doi:10.1111/aos.70083)
Supplement: Supplementary file 2 — Appendix S2. [file AOS-104-e503-s001.docx]

|  | Novice - Group 1 | Junior - Group 2 | Senior- Group 3 | Experts - Group 4 | Other surgical specialties - Group 5 | p-value |
| --- | --- | --- | --- | --- | --- | --- |
| Objective assessment, median(IQR) |  |  |  |  |  |  |
| Task 1 - Score Run 1 | 5.00 (4.00-6.00) | 6.00 (4.75-6.00) | 5.00 (4.00-7.00) | 6.00 (5.00-7.00) | 3.00 (2.75-3.00) | ***0.007**** |
| Task 1 - Score Run 2 | 5.00 (5.00-5.00) | 5.00 (3.75-6.00) | 5.00 (4.00-7.00) | 6.00 (6.00-7.00) | 4.00 (2.75-5.00) | ***0.031**** |
| Task 2 - Score Run 1 | 1.00 (0.00-4.00) | 4.00 (4.00-4.00) | 4.00 (1.00-4.00) | 4.00 (4.00-4.00) | 3.00 (2.50-4.00) | ***0.015**** |
| Task 2 - Score Run 2 | 3.00 (-1.00-4.00) | 4.00 (4.00-4.00) | 4.00 (4.00-4.00) | 4.00 (4.00-4.00) | 4.00 (3.00-4.00) | *0,093* |
| Task 3 - Score Run 1 | 1.00 (1.00-2.00) | 1.00 (1.00-2.00) | 1.00 (1.00-2.00) | 1.00 (1.00-2.00) | 1.00 (1.00-1.00) | *0,626* |
| Task 3 - Score Run 2 | 2.00 (1.00-2.00) | 2.00 (1.75-2.00) | 1.00 (1.00-2.00) | 2.00 (2.00-2.00) | 1.00 (1.00-1.25) | *0,054* |
| Task 4 - Score Run 1 | 5.00 (5.00-5.00) | 5.00 (4.75-5.00) | 5.00 (4.00-5.00) | 5.00 (5.00-5.00) | 4.00 (4.00-4.25) | ***0.036**** |
| Task 4 - Score Run 2 | 5.00 (5.00-5.00) | 5.00 (4.75-5.00) | 5.00 (5.00-5.00) | 5.00 (5.00-5.00) | 5.00 (4.75-5.00) | *0,948* |
| Task 5 - Score Run 1 | 3.00 (1.00-3.00) | 3.00 (1.50-3.00) | 1.00 (0.00-3.00) | 3.00 (0.00-3.00) | 1.00 (0.00-2.25) | *0,237* |
| Task 5 - Score Run 2 | 3.00 (3.00-3.00) | 2.00 (0.75-2.25) | 3.00 (0.00-3.00) | 3.00 (0.00-3.00) | 1.50 (-0.75-2.25) | *0,293* |
| Task 6 - Score Run 1 | 4.00 (-5.00-5.00) | 4.50 (1.25-5.25) | 4.00 (3.00-5.00) | 5.00 (5.00-6.00) | -1.00 (-5.00-3.50) | ***0.054**** |
| Task 6 - Score Run 2 | 4.00 (-5.00-5.00) | 4.50 (3.75-5.25) | 5.00 (4.00-5.00) | 6.00 (5.00-6.00) | -1.00 (-5.25-4.25) | ***0.007**** |
| Task 7 - Score Run 1 | -3.00 (-6.00- -2.00) | -3.00 (-5.25- -3.00) | -3.00 (-5.00- -3.00) | -3.00 (-6.00-0.00) | -3.00 (-6.00- -3.00) | *0,577* |
| Task 7 - Score Run 2 | -3.00 (-6.00- -2.00) | -3.00 (-3.00- -1.50) | -3.00 (-6.00- -2.00) | -2.00 (-3.00- 1.00) | -3.00 (-3.75- -3.00) | *0,285* |
| Task 8 - Score Run 1 | 2.00 (2.00-3.00) | 2.00(1.75-2.25) | 2.00 (2.00-3.00) | 2.00 (1.00-3.00) | -1.00 (-5.00-3.00) | *0,951* |
| Task 8 - Score Run 2 | 3.00 (2.00-3.00) | 2.50 (2.00-3.00) | 2.00 (1.00-2.00) | 3.00 (3.00-3.00) | -2.00 (-4.00-2.00) | *0,143* |
| Task 9 - Score Run 1 | 1.00 (1.00-2.00) | 1.00 (1.00-1.00) | 1.00 (1.00-1.00) | 1.00 (1.00-2.00) | 1.00 (1.00-1.00) | *0,26* |
| Task 9 - Score Run 2 | 1.00 (1.00-2.00) | 2.00 (1.00-2.00) | 2.00 (2.00-2.00) | 2.00 (2.00-2.00) | 1.00 (0.75-1.00) | ***0.01**** |
